# Preliminary ERP evidence of the impact of loneliness on Stroop interference for socio-emotional stimuli

**DOI:** 10.3389/fnins.2025.1602325

**Published:** 2025-12-10

**Authors:** Maria Arioli, Carlotta Maiocchi, Zaira Cattaneo, Claudia Gianelli, Nicola Canessa

**Affiliations:** 1 Department of Human and Social Sciences, University of Bergamo, Bergamo, Italy; 2 IUSS Cognitive Neuroscience (ICoN) Center, Scuola Universitaria Superiore IUSS, Pavia, Italy; 3 Department of Clinical and Experimental Medicine, University of Messina, Messina, Italy; 4 Istituti Clinici Scientifici Maugeri IRCCS, Cognitive Neuroscience Laboratory of Pavia Institute, Pavia, Italy

**Keywords:** emotional Stroop task, EEG, loneliness, social concepts, attention, intervention, cognitive control

## Abstract

Growing evidence highlights the adverse clinical effects and societal implications of loneliness, i.e., the negative feeling associated with a perceived discrepancy between desired and existing social connections. To further understand the implicit attentional and cognitive control processes associated with loneliness, we used electroencephalography (EEG) and event-related potentials (ERPs) to investigate the relationship between loneliness levels and brain activity underlying attentional capture in a socio-emotional Stroop task. In keeping with previous reports of three-stage processing of socio-emotional words, positive–negative valence and social-individual content of word stimuli were reflected in the amplitude of ERP components associated with high-order perceptual processing and preliminary emotional decoding (P200), emotional and semantic processing (P300), and interference suppression (N450). In the later stage, the differential N450 amplitude associated with processing socially negative compared with control-neutral stimuli was negatively correlated with self-perceived loneliness levels. This finding suggests that social negative and neutral stimuli are processed more dissimilarly at higher levels of self-perceived loneliness, possibly due to increased hypervigilance toward negative social cues, like those associated with rejection or exclusion. By elucidating the neural mechanisms underlying the effects of loneliness on socio-cognitive processing, these findings provide novel insights that can guide future research and inform the development of innovative therapeutic interventions that target the consequences of perceived social disconnection.

## 1 Introduction

The emotional Stroop task (EST) is a variant of the original word-color Stroop task (Stroop, 1935), requiring participants to name the ink color of words with either emotional or neutral semantic content (Ben-Haim et al., 2016). While in the neutral condition, words lack valence and are characterized by a moderate level of arousal (e.g., “table”), the emotional condition includes words with positive or negative emotional valence and high arousal (e.g., “murder”). The well-established *emotional Stroop effect (ESE)* reflects longer latencies to name the ink color of emotionally negative, compared with neutral, words (Ben-Haim et al., 2016; Song et al., 2017). These tasks allow for the detection of individual susceptibility to different types of cognitive conflicts. The incongruent condition of the original Stroop task entails a mismatch between visual and semantic features, i.e., the ink color and the semantic content of the word, requiring participants to inhibit the distracting semantic information to respond correctly (MacLeod, 1991; Parris et al., 2022). Instead, EST performance reflects the ability to inhibit the larger automatic attentional capture elicited by emotionally negative, compared with neutral, word meaning (McKenna and Sharma, 2004; for a different account, see Algom et al., 2004; Ben-Haim et al., 2014). At the neurophysiological level, emotional interference during word processing has been linked to event-related potential (ERP) components, reflecting partially distinct processing stages. Early perceptual processes have been associated with the modulation of the N170 and P200 (Imbir et al., 2021a, 2021b; Liu et al., 2023), whereas later discriminative responses often emerge around the P300, which is sensitive to emotional salience and motivational relevance (Imbir et al., 2021a, 2021b; Xue et al., 2016). At later latencies, emotional Stroop paradigms frequently report an enhanced N400/N450 response (∼350–500 ms), classically associated with semantic integration and expectancy violation (e.g., Lau et al., 2008) but also interference suppression and conflict monitoring in affective and cognitive Stroop tasks (e.g., Heidlmayr et al., 2020; Imbir et al., 2021a, 2021b). Importantly, N400 amplitudes are known to vary with stimulus frequency, predictability, and contextual mismatch (Kutas and Federmeier, 2011; Rabovsky et al., 2018), but also with emotional content, particularly when negative words compete for attentional resources (Citron, 2012; Kissler et al., 2007).

The EST has been widely employed to assess emotional biases in attentional capture across healthy (e.g., Arioli et al., 2021), subclinical (e.g., Kamboureli and Economou, 2023), and clinical (e.g., Ros et al., 2023) populations, and the extent to which their sensitivity to selective semantic categories is shaped by individual and/or contextual factors (Williams et al., 1996).

In this respect, growing evidence highlights the effect of *loneliness*, i.e., the distress associated with the subjective experience of a discrepancy between desired and existing social relationships (Peplau and Perlman, 1982; Lim et al., 2020). Significant associations have been reported between the degree of self-perceived loneliness and various physical and mental outcomes, including cardiovascular disorders (Valtorta et al., 2016), dementia (Carbone et al., 2022; Kuiper et al., 2020), mood disorders (Giacco, 2023), suicidal ideation (McClelland et al., 2020), and even greater mortality (Holt-Lunstad et al., 2015). The COVID-19 pandemic has increased interest both in the adverse consequences of loneliness (Pai and Vella, 2021) and its neurocognitive precursors. Concerning the latter aspect, behavioral studies suggest that loneliness shapes the attentional processing of social stimuli through hypervigilance to *negative social* information (Cacioppo et al., 2016). For instance, individuals who perceive themselves as lonely exhibit heightened sensitivity to painful expressions (Yamada and Decety, 2009), reject body postures (Bangee et al., 2014), and are more prone to experiencing negative feelings such as hostility and alertness (Meng et al., 2020).

It is therefore unsurprising that the degree of self-perceived loneliness additionally upregulates the emotional interference effect, as tracked by a larger attentional capture by negative emotional stimuli (Shin and Kim, 2019). The neurobiological basis of this modulation has been explored by coupling electroencephalography (EEG) with a socio-emotional Stroop, differentiating between the effects of social and non-social positive and negative emotional words (Cacioppo et al., 2015). Results showed that the degree of loneliness is reflected in stronger responses of visual areas to negative social (vs. negative non-social) words, suggesting implicit hyperattention during their processing by lonely individuals. Similarly, the degree of loneliness was found to reflect in enhanced attention to *negative emotional* distractors, stronger theta and beta activity in temporo-parietal regions (Grennan et al., 2021), and faster responses to negative emotions, compared with neutral stimuli, in lonely individuals (Du et al., 2022). This combined evidence supports the evolutionary theory of loneliness (Cacioppo et al., 2014), suggesting that feelings of social isolation increase attentional focus on negative social stimuli and their processing as potential threats, thereby increasing vigilance in the social environment as an unintentional strategy of self-preservation (Cacioppo and Cacioppo, 2018).

This hypothesis is supported by a recent meta-analysis of functional and structural magnetic resonance imaging data suggesting that, in lonely individuals, heightened bottom-up attentional bias toward socio-emotional negative stimuli is associated with compensatory top-down cognitive control mechanisms (Wong et al., 2022). However, this promising model is only partially supported by the available EEG evidence of no compensatory mechanism for this attentional bias in lonely individuals (Cacioppo et al., 2015; Grennan et al., 2021). This gap might reflect the use of basic emotional—rather than social—stimuli (as in Grennan et al., 2021 and Du et al., 2022; for discussions, see Luo and Shao, 2023) or the need for additional metrics of brain activity. While previous EEG studies have either analysed microstates (Cacioppo et al., 2015) or cortical oscillations (Grennan et al., 2021), ERPs are considered an ideal neural metric of emotion-attention interactions (Ding et al., 2017; Schupp et al., 2006).

We therefore performed an EEG-ERP study to investigate the neural processing of socio-emotional words and the effect of loneliness on attentional capture by social negative stimuli in 34 healthy young individuals engaged in a socio-emotional Stroop task (Arioli et al., 2021). Based on previous evidence from both lonely (Du et al., 2022) and non-lonely (Ben-Haim et al., 2016) individuals, we expected to observe slower responses to negative social stimuli compared to neutral ones in individuals with lower self-reported loneliness and a reversal of this pattern at the highest loneliness levels. Moreover, based on the available meta-analytic evidence (Wong et al., 2022), at the neural level, we predicted that loneliness influences the three stages of emotional word processing (Liu et al., 2023), involving both an early bottom-up attentional bias toward negative social stimuli and a later compensatory top-down cognitive control mechanism sustaining task performance.

## 2 Materials and methods

### 2.1 Participants

Thirty-four healthy volunteers (21 females; mean age = 23 years, standard deviation (SD) = 3.00, range = 20–35) were recruited to participate in the experiment. The sample size was determined based on a previous power analysis by Imbir et al. (2021a), where an expected η^2^ of 0.10–0.15 for the emotional Stroop effect on EEG signals resulted in a minimum of 18 participants at 0.95 power. To ensure adequate statistical power and compensate for potential data loss, we recruited 34 participants (with 4 excluded for poor behavioral performance and 3 for poor EEG quality). All participants were right-handed native Italian speakers with normal or corrected-to-normal vision. A history of neuropsychiatric conditions that may affect EEG recording (e.g., epilepsy) or substance abuse was considered an exclusion criterion. Participants provided written informed consent to participate in the experiment, which was developed in accordance with the latest version of the Declaration of Helsinki and approved by the Ethics Committee of ICS Maugeri Pavia.

### 2.2 Experimental design and procedure

Participants performed a socio-emotional Stroop task developed in our lab and previously coupled with functional Magnetic Resonance Imaging (fMRI; Arioli et al., 2021). This task included four primary target conditions, each comprising 16 high-arousal Italian words with varying semantic content (individual emotional vs. social emotional) and valence (positive vs. negative). The resulting conditions were defined as follows: IP (individual positive), IN (individual negative), SP (social positive), and SN (social negative). The additional control condition (CC) included 64 neutral words. To maintain consistency across studies while minimizing participants’ fatigue and maximizing data quality, the number of trials was kept identical to the fMRI design. Details of the procedures for word classification and word selection have been described by Arioli et al. (2021).

**Figure 1:**
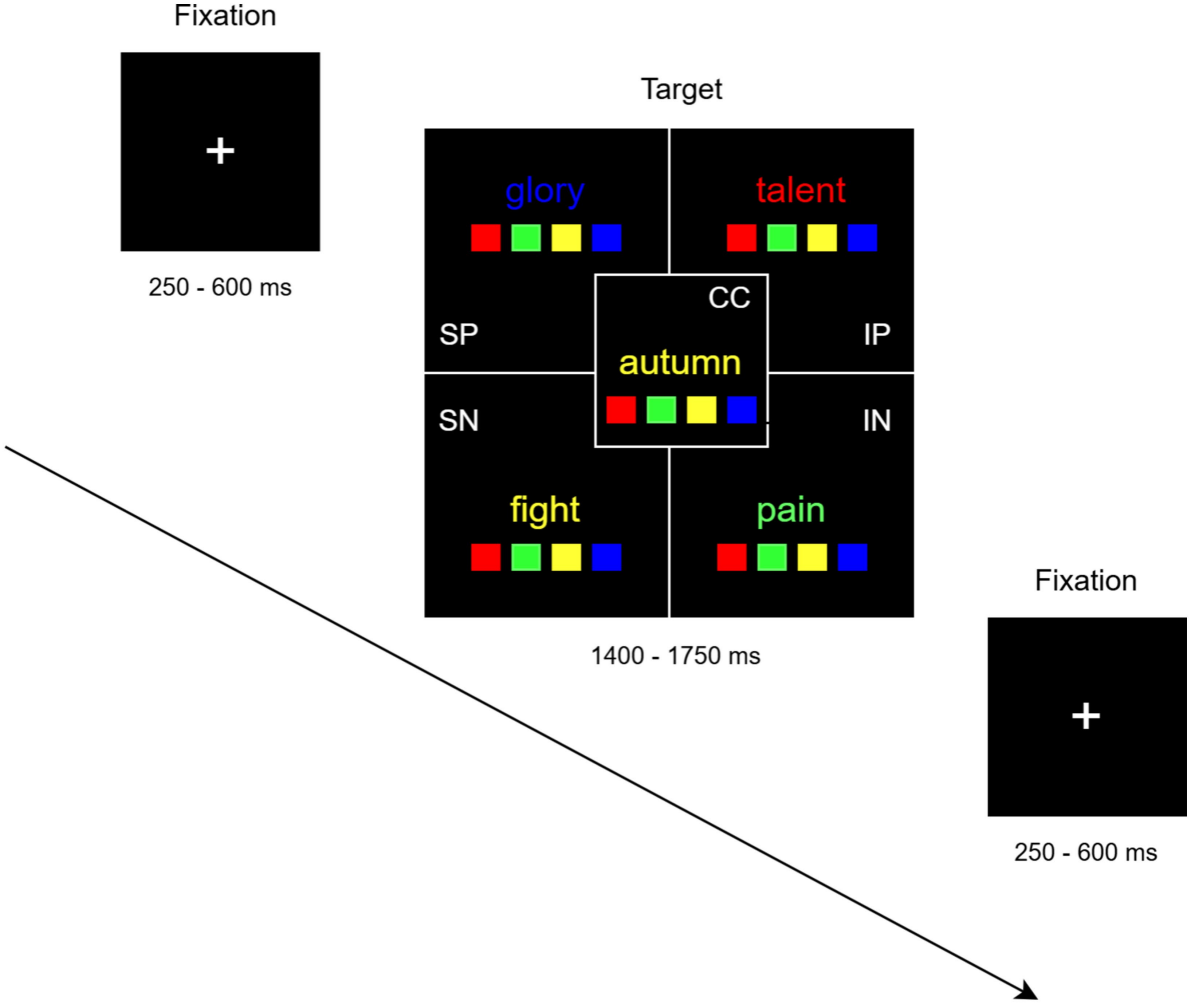
Experimental procedure. During each trial, a word was displayed on a black background for an average duration of 1,575 ms (ranging from 1,400 to 1750 ms), followed by a varying inter-stimulus interval (ISI) (ranging from 250 to 600 ms) during which a white fixation cross was shown. The figure reports examples of single trials for the four target conditions of interest, i.e., individual positive (IP), individual negative (IN), social positive (SP), social negative (SN), and control condition (CC). Words were printed in red, yellow, green, or blue, with each color occurring equally often across conditions. The labels were reported for explanatory purposes and were not shown during task execution.

Participants were provided with standardized instructions and completed a training session before EEG data collection. The training session was carried out immediately before the EEG session and included 16 trials representative of all experimental conditions. They were instructed to identify the ink color of each word as quickly and accurately as possible. Words were printed in red, yellow, green, or blue, with each color appearing equally across conditions. Responses were recorded using four buttons corresponding to the spatial positions of colored panels displayed below the word (Figure 1). Participants responded using the four fingers of their right hand (index to pinkie). Stimuli were presented, and responses were collected using presentation software (www.neurobs.com).

During each trial, a word was displayed on a black background for an average duration of 1,575 ms (ranging from 1,400 to 1750 ms), followed by a jittered inter-stimulus interval (ISI; ranging from 250 to 600 ms), during which a white fixation cross was shown. Words were always presented at the centre of the horizontal plane. Instead, to discourage participants from focusing on a predetermined word section, their vertical offset was trial-wise varied among 8 positions ranging between 20 and 125 pixels from the centre, in steps of 15. Following Ben-Haim et al. (2016), words were grouped into blocks, each consisting of either 8 neutral words (CC) or 8 words from a single target condition (SN, SP, IN or IP), resulting in a block duration of 16 s. The stimulus duration was consistent across all conditions, and the word order within each block was individually randomized for every participant.

Across two EEG runs, a total of 16 neutral blocks and 4 blocks per target condition were presented. Each run comprised 128 trials, resulting in a total of 256 trials (32 trials per target condition plus 128 neutral trials), with each word displayed twice. To prevent the consecutive presentation of two target blocks, the target and neutral blocks were randomly alternated within each run. Additionally, the word colors for each condition were counterbalanced across blocks within a run, and the order of runs was counterbalanced across participants.

We assessed participants’ self-perception of social isolation using the R-UCLA Loneliness Scale (Russell, 1996), a well-established instrument for measuring loneliness (Cacioppo and Cacioppo, 2018). This questionnaire yielded a total score between 20 and 80 points. Based on their scores, participants were classified into the “high loneliness” (>41) and “low loneliness” (<=41) groups.

### 2.3 EEG recording

Continuous electroencephalography (EEG) data were recorded from 64 active channels using the G.tec system (G.tec Medical Engineering GmbH) with Cz as the online reference electrode during acquisition. The data were then re-referenced to a common average reference during the preprocessing stage. The inter-electrode impedance was maintained below 10 kΩ throughout the recording. The electrophysiological data were imported into MATLAB and processed using the EEGLAB toolbox (version 2022.0; Delorme and Makeig, 2004) and custom scripts. The EEG signal was downsampled to 600 Hz for data reduction, and a high-pass filter at 1 Hz was applied. For statistical analyses, epochs were defined to (a) include a 1,000 ms pre-stimulus baseline and (b) extend 2000 ms post-stimulus onset. These epochs were visually examined to exclude segments with artefact-related sources of noise. Independent Component Analysis (ICA) weights were then computed using the RUNICA algorithm implemented in EEGLAB. The components were classified using the ICLabel plugin, and those associated with eye blinks, muscle movements, and channel and noise artefacts were automatically removed. After offline artefact rejection, ERPs were computed—only for trials with correct responses—focusing on the time window from −200 to 800 ms, with 0 ms marking the onset of the target stimulus. Electrode selection and time windows were defined based on previous evidence from an emotional Stroop task (Imbir et al., 2021b; Shen et al., 2013). Since these studies suggested that the ERP components of interest (P200, P300, N450) involve fronto-medial and posterior-parietal regions, analyses focused on data from electrodes Fz, F3, F4, FCz, Cz, C3, C4, P3, P4, Pz, PO3, and PO4, within 160–250 ms (P200), 250–400 ms (P300), and 350–550 ms (N450).

### 2.4 Statistical analyses

We analysed behavioral data with the R statistical package (R Core Team, 2024), using parametric statistics after verifying that both response accuracy and RTs followed a Gaussian distribution. We performed a preliminary quality-check stage, with the exclusion criterion being an accuracy below two standard deviations from the group mean in at least one condition.

To address the neural processing of socio-emotional words, we ran a repeated-measures ANOVA with condition (SP, SN, IP, IN, CC) as a within-subject factor, and, in a second step, we added the UCLA loneliness score as a covariate. In the case of significant main effects, post-hoc paired t-tests were performed. To align statistical analyses with the factorial structure of the task, and following previous related studies (e.g., Arioli et al., 2021; Ben-Haim et al., 2016), we also performed a 2 × 2 repeated-measures ANCOVA with valence (positive vs. negative) and content (social vs. individual) as within-subject factors, and, in a second step, we added loneliness score as a covariate. To directly assess the relationship between emotional interference and loneliness, we performed correlational analyses to examine the associations between RT difference and UCLA loneliness scores.

We applied the same analytical structure to the EEG data. For each ERP component of interest, the mean amplitude (averaged across relevant electrodes and time windows) served as the dependent variable, with condition as a within-subject factor and loneliness as a covariate. In addition, we performed correlational analyses between condition-specific ERP amplitudes and RTs to explore potential functional associations between neural and behavioral markers of emotional interference. We tested for violations of sphericity using Mauchly’s test and applied the Greenhouse–Geisser correction where appropriate. Time and amplitude values are reported in milliseconds and microvolts, respectively.

## 3 Results

We excluded four participants because of low accuracy (>2 SD below the mean for at least one condition) and another three participants because of poor EEG data quality. Of the remaining 27 participants (14 females; mean age = 23 years, SD = 3), 16 (9 females; mean age = 23 years, SD = 4) and 11 (5 females; mean age = 22.6 years, SD = 2.07) reported “low loneliness” and “high loneliness” scores, respectively, on the UCLA loneliness scale.

### 3.1 Behavioral results

Following Ben-Haim et al. (2016), we analysed RTs considering only trials with correct responses. A repeated measures ANOVA, with condition (SP, SN, IP, IN, CC) as a within-subject factor, revealed a marginally significant main effect of condition (*F*(4,104) = 2.43, *p* = 0.052). Post-hoc comparisons indeed highlighted a marginally significant difference between the SN condition (mean RT = 647 ms, SD = 89) and the CC condition (mean RT = 670 ms, SD = 102) (t(26) = 2.96, *p* = 0.064; Bonferroni corrected). This effect disappeared when loneliness was included as a covariate in the model [*F*(4,100) = 1.29, *p* = 0.28]. The 2×2 repeated measures ANOVA revealed a significant interaction between content (social vs. individual) and valence (positive vs. negative) [*F*(1,26) = 10.10, *p* = 0.004]. Post-hoc tests showed a marginal effect of content within the negatively valenced stimuli, with shorter RTs for social (SN: mean RT = 647 ms, SD = 89) compared to individual (IN: mean RT = 670 ms, SD = 102) content [t(26) = 2.72, *p* = 0.052], but not for positively valenced ones (t(26) = 0.82, *p* = 0.84). No significant main effects of content [F(1,26) = 1.31, *p* = 0.26] or valence [F(1,26) = 0.13, *p* = 0.72] were observed. When including loneliness as a covariate, we observed neither a significant main effect of content (*F*(1,25) = 0.84, *p* = 0.37), valence [F(1,25) = 0.31, *p* = 0.58], or loneliness [*F*(1,25) = 0.98, *p* = 0.33], nor an interaction between valence × loneliness [F(1,25) = 2.81, *p* = 0.10], content × loneliness [F(1,25) = 0.46, *p* = 0.50] or valence × content × loneliness [*F*(1,23) = 0.03, *p* = 0.85].

**Figure 2:**
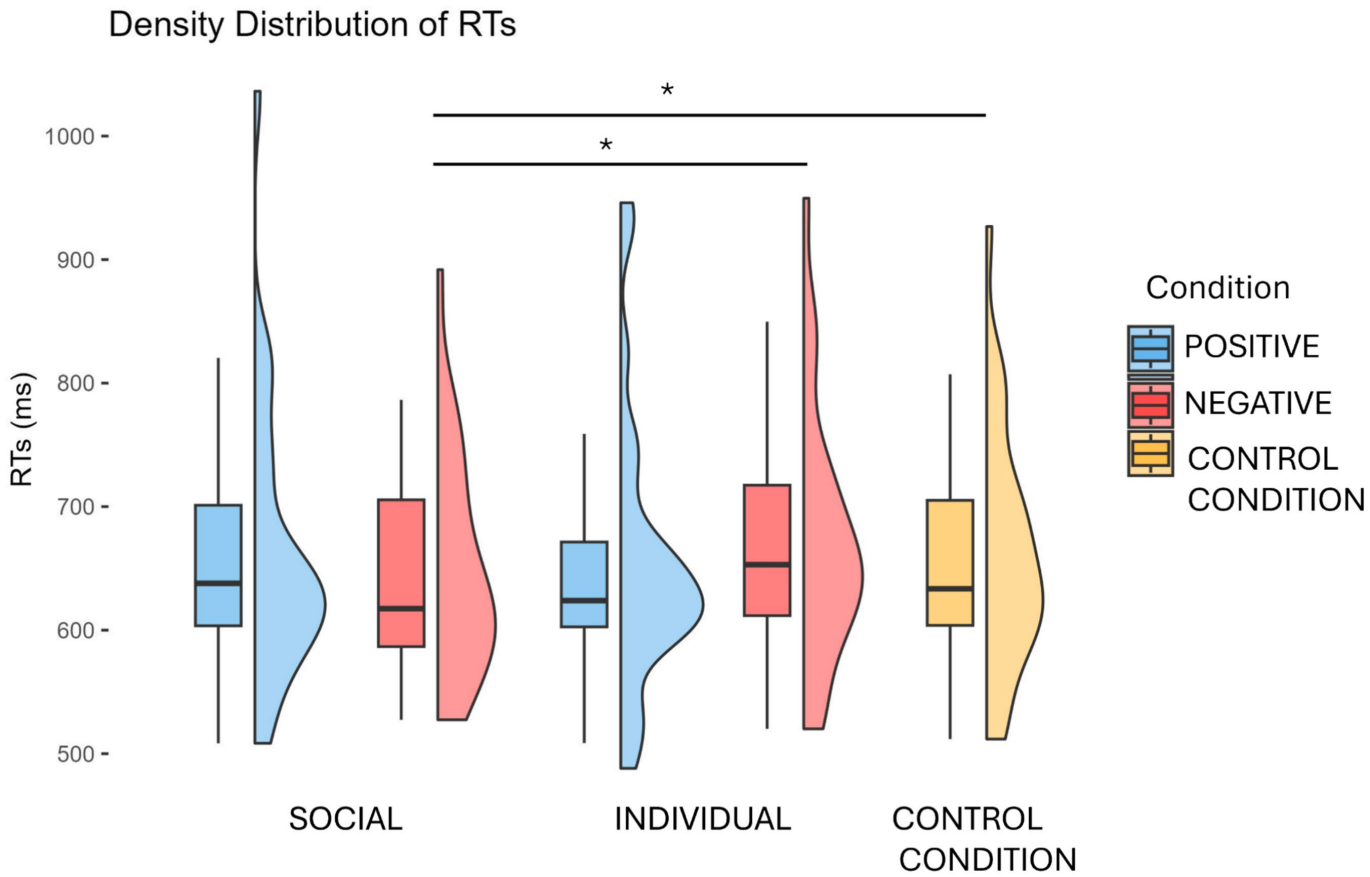
Behavioral results (RTs). For each condition, boxplots depict the median value and interquartile range of RTs (with error bars indicating the standard error of the mean (SEM)), while violin plots show their distribution. Asterisks denote significant differences from post-hoc comparisons with Bonferroni correction.

Spearman’s correlations between individual RT differences and loneliness scores did not show any significant effect (all *p* > 0.05). (Figure 2).

### 3.2 ERP results

As described above, based on prior studies (Imbir et al., 2021a; Shen et al., 2013; Zhang et al., 2014), ERP analyses focused on specific electrodes (F1, F2, F3, F4, FCz, Fz, C3, C4, P3, P4, PO3, PO4, and Pz). In distinct models, we analysed either the effect of condition or the effects of content and valence, alongside their association with loneliness, on components P200, P300, and N450 (see ERP boxplot in Supplementary material).

#### 3.2.1 P200 component

We defined the P200 component as occurring across the fronto-medial electrodes within the 160–250 ms time interval. Significant P200 modulations were found across F4, Fz, and FCz electrodes, and, in particular, a one-way repeated measures ANOVA, with loneliness as a covariate, indicated a significant effect of condition on P2 amplitudes [*F*(4,100) = 3.84, *p* = 0.006]. Post-hoc contrasts with Bonferroni correction showed significantly higher P200 amplitudes for individual positive stimuli than for control stimuli [IP-CC t(25) = 3.15, *p* = 0.04]. Further analyses revealed no significant correlation between the differential P200 amplitude in IP compared to CC and loneliness (*ρ* = −0.19, *p* = 0.35) or RTs (ρ = −0.28, *p* = 0.16). The 2 × 2 repeated measures ANOVA, with content and valence as within factors and loneliness as a covariate, highlighted a significant effect of valence [*F*(1,25) = 10.26, *p* = 0.004], with no main effect of content [F(1,25) = 0.03, *p* = 0.8] or interaction between content and valence [F(1,25) = 0.58, *p* = 0.46]. Moreover, loneliness had no effect on P200 amplitudes [F(1,25) = 0.44, *p* = 0.51].

#### 3.2.2 P300 component

**Figure 3:**
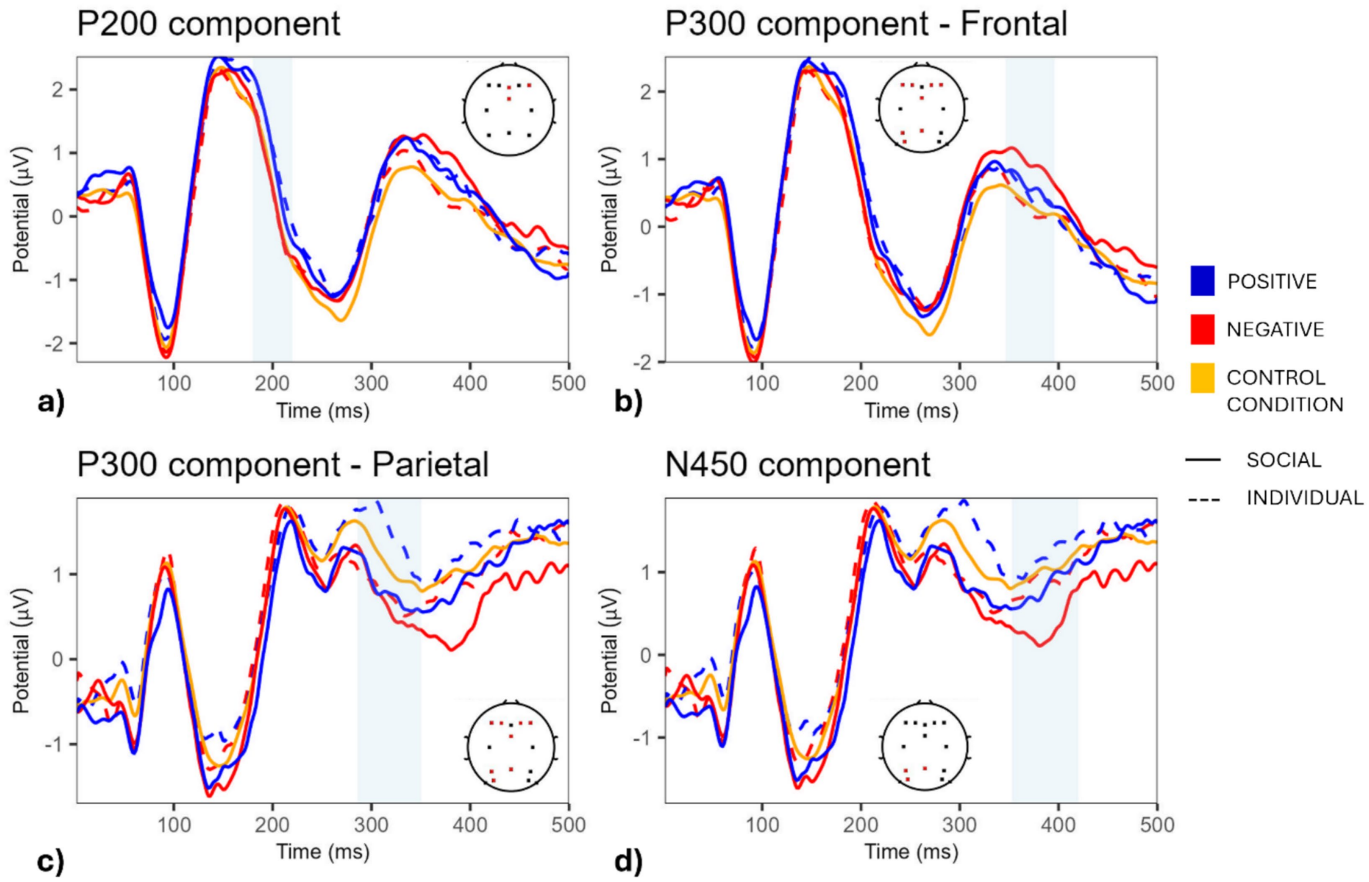
ERP component analysis across conditions.The figure depicts ERPs for the components of interest in association with positively (blue)- and negatively (red)-valenced individual (dashed lines), social (solid lines), and neutral (yellow) word stimuli. The P200 component is shown at fronto-medial electrodes, highlighting the 160–250 ms time window **(a)**; the P300 component is activated both in a frontal cluster in the 350–390 ms time interval **(b)**; in a parietal cluster in the 280–350 ms time interval **(c)**; and the N450 component involves midline and posterior parietal electrodes within the 350–550 ms time window **(d)**.

We found significant P300 component amplitudes over the frontal and parietal electrodes in the 280–390 ms time interval. A repeated measures ANOVA was performed to investigate their modulation by condition (SP, SN, IP, IN, and CC), loneliness score, and their interaction. This analysis highlighted a significant effect of condition on P3 amplitudes in the frontal cluster (F1-F2-F3-F4-FCz) (*F* = 2.89, *p* = 0.026) in the 350–390 ms time interval, with post-hoc contrasts revealing a significantly higher amplitude in SP compared with CC (*p* = 0.0039). Further analyses revealed no significant correlation between the differential P300 amplitude in the SP vs. CC condition and loneliness (ρ = −0.05, *p* = 0.80) or RTs (*ρ* = 0.11, *p* = 0.57). A 2 × 2 repeated measures ANCOVA highlighted no significant main effect of content (social vs. individual) or valence (positive vs. negative), nor their interaction, on P300 amplitudes. A marginal, but not significant, interaction between loneliness and content was found [F(1,25) = 3.035, *p* = 0.09] (Figure 3).

Concerning the parietal cluster, we observed a significant effect of condition on P300 amplitudes (*F* = 5.69, *p* < 0.001) between 280 and 350 ms, with post-hoc analyses showing significant differences between SN and IP (p = 0.04), as well as IP and IN (p = 0.02). A 2 × 2 repeated measures ANCOVA additionally highlighted significant main effects of content (i.e., higher P300 amplitude for individual compared with social content; *F* = 4.63, *p* = 0.041) and valence (i.e., higher P300 amplitude for positive compared with negative stimuli; *F* = 11.25, *p* = 0.003) and a marginal content x valence interaction (*F* = 3.62, *p* = 0.068). The latter finding reflected a significant difference between Individual Positive and Social Negative words [t(25) = 3.17, *p* = 0.02] and between Individual Positive and Individual Negative words [t(25) = 3.38, *p* = 0.012] (Figure 3). However, no significant effect of loneliness was observed.

#### 3.2.3 N450 component

**Figure 4:**
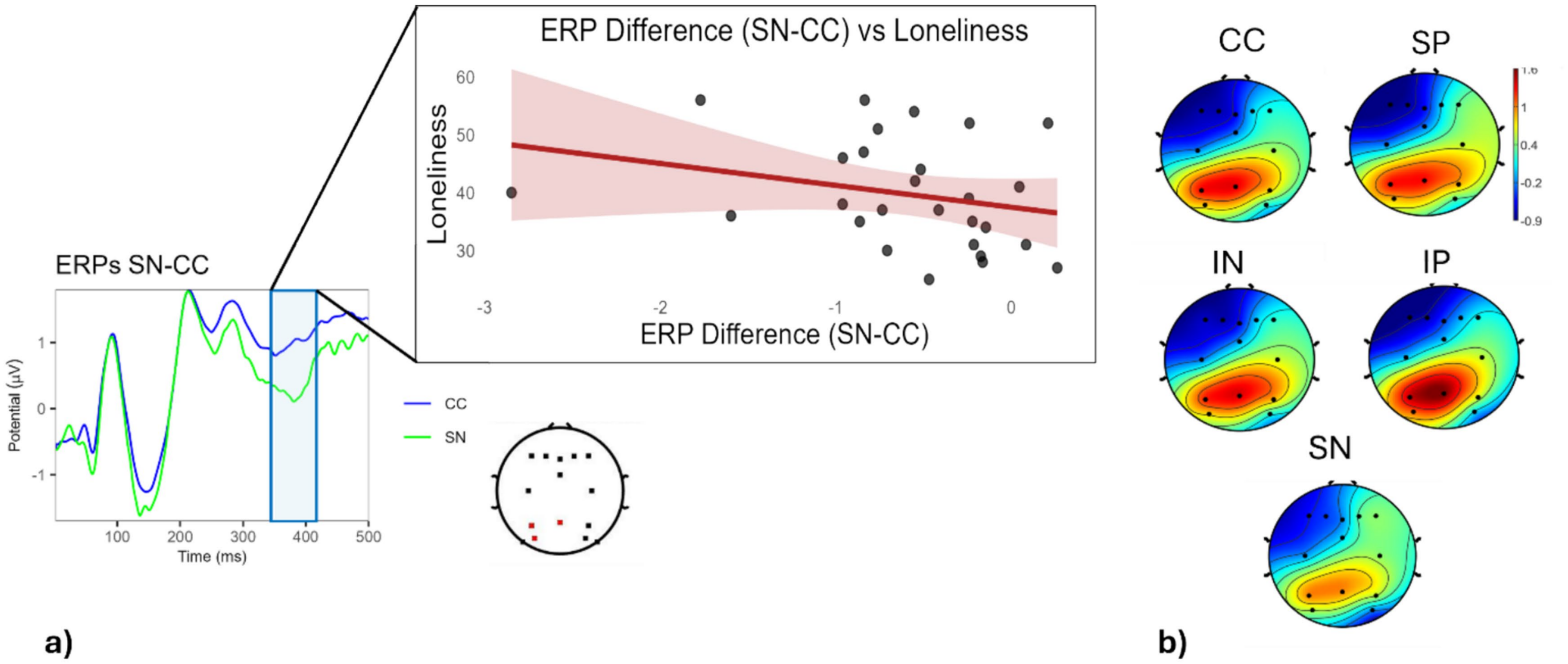
N450 SN-CC ERP component analysis and correlation with loneliness. **(a)** The left panel depicts ERP waveforms for the SN and CC conditions at electrodes Pz, P3, and PO3, highlighting a significant 350–550 ms time window. The middle panel shows the negative correlation between loneliness and the differential N450 amplitude in SN vs. CC. **(b)** The topographic maps display the scalp distribution of the N450 component across conditions within the 350–550 ms time range.

We evaluated the N450 component across midline and posterior parietal electrodes to examine further potential content/valence-related effects within the 350–550 ms time window post-stimulus onset. A one-way repeated-measures ANOVA, followed by cluster-based permutation correction (N = 1,000 permutations, *p* < 0.05), revealed a significant effect of condition on ERP amplitude at electrodes Pz, P3, and PO3 (*F*(4,100) = 4.97, *p* = 0.001) between 350 and 420 ms. Post-hoc comparisons with Bonferroni correction revealed a significantly more negative amplitude in the SN condition compared with both the IP (*p* = 0.0046) and CC (*p* = 0.0045) conditions. Further analyses showed a significant negative correlation between loneliness and differential N450 amplitude in SN than CC (*ρ* = −0.41, *p* = 0.035). This joint evidence highlights an increased differentiation in N450 responses toward social negative words, compared with control stimuli, at higher levels of loneliness. However, no significant relationship was found between the differential N450 amplitude and RTs (*ρ* = −0.04, *p* = 0.84) (Figure 4). When modeling a 2 × 2 ANCOVA, we observed significant effects of both content [*F*(1,25) = 7.46, *p* = 0.01] and valence [*F*(1,25) = 10.45, *p* = 0.003]. Specifically, a more negative N450 amplitude was found for social (compared with individual) content and negative (compared with positive) valence. However, no interaction between content and valence was found [F(1,25) = 0.003, *p* = 0.99].

## 4 Discussion

To provide novel insights into the neural processing of socio-emotional words and the effect of loneliness on attentional and cognitive control processes (Wong et al., 2022), we investigated its relationship with changes in brain ERPs while performing a socio-emotional Stroop task with positively and negatively valenced words (Arioli et al., 2021). We found evidence for a three-stage neural processing of socio-emotional Stroop, involving the P200, P300, and N450 components. Contrary to our expectations, the individual loneliness level appeared to be associated with the latest stage of processing social negative words rather than early neural responses, possibly suggesting a later hypervigilance to negative social stimuli in our participants.

The behavioral finding of faster RTs to social negative (SN) than neutral (CC) words was unexpected in light of the typical ESE (i.e., longer RTs for negative vs. neutral words) in the general healthy population. Our methodological approach, based on interleaved “emotional” and “control” blocks, may have attenuated the ESE, which is typically calculated as the differential performance between the emotional block and the *first* neutral block (Ben-Haim et al., 2016). Consistent with this interpretation, one of the few studies to report *shorter* RTs for negative words compared with neutral words (Imbir et al., 2021a) employed an experimental design similar to that used in the present study. Notably, this effect was no longer observed when loneliness was included as a covariate. While this finding strengthens our hypothesis that ESE is modulated by the degree of self-perceived loneliness, the fact that the latter was not related to the difference in RTs between SN and CC prevents a clear overview of the effect of loneliness on attentional capture by emotional and/or social stimuli. This complex picture may also depend on the relatively small sample size, mainly young university students with moderately high levels of loneliness (maximum score of 56 out of 80), thus limiting the possibility of studying the effects of extremely high loneliness.

Regarding the association between behavioral and neurophysiological findings, our results are consistent with prior evidence from studies employing the emotional Stroop task (Imbir et al., 2021a, 2021b; Xue et al., 2016). In particular, the effects of word valence and content supported a three-stage processing of socio-emotional words (Liu et al., 2023; Zhang et al., 2014) involving components underlying perceptual processing (P200), emotional salience processing (P300), and monitoring and interference suppression (N450). The latter processing stage was additionally modulated by the degree of self-reported loneliness, which was negatively correlated with the differential N450 amplitude associated with processing social negative (SN) and control words (CC).

The initial involvement of P200, originating from fronto-medial areas, is suggestive of an early perceptual processing of Stroop words (e.g., Imbir et al., 2021b), which supports the role of this positive component in preliminary emotional encoding of the attended stimuli (Paulmann et al., 2013). Indeed, in keeping with previous related evidence on emotional word processing (Kanske and Kotz, 2007; Schapkin et al., 2000; Xie et al., 2014), word valence exerted a significant modulation of the P200 amplitude, with positively valenced stimuli eliciting a more positive amplitude compared with both negative and control stimuli. This early valence effect might underpin an “intrinsic pleasantness” check, which would fit both with the “component process model” of emotion (Scherer, 2009; Keuper et al., 2013) and with the assumption of a positivity bias (Hoorens, 2021; Kuchinke et al., 2005; but see evidence in favor of the negativity bias, Smith et al., 2003).

In the subsequent stage, an effect of word valence was also observed in the P300, a parietal and frontal positive component previously associated with emotional processing (Ding et al., 2017). This result aligns with previous reports of a higher P300 amplitude for positive than negative words (Conroy and Polich, 2007; Zhang et al., 2014), which might reflect a stronger modulation of processes susceptible to positivity bias, such as attentional capture, evaluation, decision-making, and/or memory encoding (Herbert et al., 2006). Interestingly, the parietal P300 was also significantly modulated by word content, with a higher P300 amplitude for individual words than for social words, confirming the effectiveness of our task in eliciting implicit processing of distinct categories of word stimuli. Previous studies have helped interpret the involvement of this component in the semantic processing of written words (Dorjee et al., 2010; Hill et al., 2005), particularly when processing social content (Funkhouser et al., 2019; Liu et al., 2021). Overall, our results support the role of P300 in emotional and semantic processing in association with the Stroop task.

Finally, for the last stage, we focused on the N450, a negative component typically associated with semantic processing (Lau et al., 2008) and interference suppression (Heidlmayr et al., 2020). In line with its previous involvement in the emotional Stroop task (Gootjes et al., 2011; Sass et al., 2010), we specifically observed a more negative N450 for SN words compared with CC words, likely indicating the facilitation of their semantic processing (Sass et al., 2010). The specificity of this finding is strengthened by a significant negative correlation between the N450 for social negative words and self-reported loneliness, reflecting a larger difference in the N450 for SN words than CC words at higher loneliness levels. Although the latency of the observed effect overlaps with that of the classic N400, its functional meaning is likely multifaceted. This ERP component is typically associated with semantic integration and expectancy violation (Kutas and Federmeier, 2011; Lau et al., 2008; Rabovsky et al., 2018); however, comparable negativities have also been linked to interference suppression in emotional Stroop tasks (Heidlmayr et al., 2020; Imbir et al., 2021a, 2021b). As lexical frequency and word length were controlled, and the target/control ratio was the same across conditions, a purely expectancy-based account of the observed differences across target words seems unlikely. Instead, the modulation of this component by valence and loneliness indicates an additional contribution from affective interference. The present findings thus suggest that late negativities in this time window may reflect the joint influence of semantic expectancy and emotional conflict processes.

In this respect, our results support and complement previous EEG evidence on the neural mechanisms underlying the cognitive consequences of loneliness (e.g., Cacioppo et al., 2015; Du et al., 2022; Grennan et al., 2021). In line with our expectations and previous studies (e.g., Cacioppo et al., 2015; Du et al., 2022), these findings indeed support the view of a deeper neural processing of social negative stimuli (i.e., hypervigilance) at higher levels of self-perceived loneliness. Influential models interpret this relationship in terms of heightened attention to negative social cues—perceived as potential threats in the lack of social support—thereby promoting increased vigilance as a possible self-preservation strategy (Cacioppo and Cacioppo, 2018). The present findings enrich this model by showing an association between loneliness and hypervigilance in the *later* neural processing stage of social negative stimuli, which highlights a possible link between previously reported neural mechanisms underlying social rejection (Lurquin et al., 2014) and social anxiety (Cao et al., 2022). This hypothesis highlights the possible existence of qualitatively different neural processes mediating the effect of loneliness on hypervigilance, i.e., an extremely rapid attentional orientation toward social negative stimuli at the highest degree of loneliness (Cacioppo et al., 2015; Du et al., 2022) and a later neural modulation at moderately high loneliness levels. Importantly, the engagement of *early* hypervigilance toward social negative stimuli at higher loneliness levels has been investigated through microstate analyses (Cacioppo et al., 2015), which, compared to ERPs, involve a precise sequence of information processing prioritizing the spatial distribution of brain activity over the temporal one (Cacioppo and Cacioppo, 2018). This methodological difference might explain the detection of temporally distinct neural mechanisms across studies, reflecting the modulation of loneliness in the processing of social negative words.

Contrary to our expectations and to the “cognitive control model” of loneliness in emotional processing (Wong et al., 2022), we did not find evidence of neurophysiological mechanisms underlying the inhibition of the affective impact of social negative words. This null finding is likely driven by the nature of the task, which entails implicit processing of emotional stimuli, as their semantic content is irrelevant to task performance. Such an experimental design appears better suited for investigating attentional processes than emotion regulation and/or cognitive control. Examining whether and how loneliness modulates cognitive control may instead require the explicit processing of social negative stimuli, such as a task requiring the regulation of emotions elicited by scenes of social rejection, possibly in combination with physiological measures [e.g., skin conductance response; Matejka et al. (2013)]. Supporting this view, previous EEG studies using the emotional Stroop task have not found evidence of inhibitory brain mechanisms differentiating lonely from non-lonely individuals (Cacioppo et al., 2015; Grennan et al., 2021).

Notably, the effects of loneliness on the N450 were only detected at the ERP level, without corresponding behavioral correlates, thus limiting the scope and interpretability of our findings. This null behavioral evidence may reflect limited statistical power and variance in a small and relatively homogeneous sample of young university students with moderately high loneliness. As previously mentioned, indeed, our conclusions should be interpreted with caution given the small sample size, which likely limited the statistical power, particularly for analyses of individual differences in ERP measures. Although our target sample size was determined a priori based on estimates from a prior study employing the same task (Imbir et al., 2021a), replication with larger and more diverse cohorts is required to corroborate our findings. In addition, the sample mostly included young university students with moderately high levels of loneliness, which may constrain the generalizability of the results to a broader or more heterogeneous population. Notwithstanding these limitations, we provided novel insights into the neurophysiological correlates of (1) a three-stage neural processing, involving the P200, P300, and N450 components, when performing a socio-emotional Stroop task and (2) hypervigilance to social negative stimuli in individuals with moderately high levels of loneliness, reflected in a deeper engagement in the third stage of word processing. These findings have multiple, multifaceted implications.

First, they provide theoretical models of loneliness and its functions. From an evolutionary perspective (Cacioppo and Cacioppo, 2018), the adverse impact of loneliness is aimed at supporting healthy social interactions and preventing possible socio-physical damage. In keeping with this view, the present evidence of neural mechanisms biasing attention toward social negative stimuli at higher levels of self-perceived loneliness supports the notion that loneliness specifically enhances sensitivity to *negatively valenced social interactions* (Cacioppo et al., 2015; Shin and Kim, 2019), including social rejection and/or exclusion, and the depth of its neurocognitive processing.

Second, and even more importantly, understanding the neurophysiological correlates of loneliness effects is essential in light of its consequences at clinical and societal levels (Lim et al., 2020). Unveiling the neural mechanisms underlying the cognitive and affective impacts of loneliness might help develop novel therapeutic strategies, such as pharmacological treatments and neurostimulation protocols (Lam et al., 2021), thereby improving the quality of life of a growing population (Leigh-Hunt et al., 2017). From this perspective, our findings suggest that individuals experiencing moderately high levels of loneliness may exhibit a later-emerging neural response underlying hypervigilance toward socially negative stimuli. This response may represent a marker of the adverse consequences of loneliness on neurocognitive processing, enabling timely interventions to prevent progression toward more severe forms of perceived social disconnection associated with well-documented and significant clinical consequences (Crowe et al., 2024).

## Data Availability

The datasets presented in this article are not readily available because the data supporting the findings of this study are available from the corresponding author on reasonable request. Requests to access the datasets should be directed to nicola.canessa@iusspavia.it.
