# Supplementary material for: Preliminary ERP evidence of the impact of loneliness on Stroop interference for socio-emotional stimuli
Source: Front Neurosci. 2025 Dec 10;19:1602325. doi: 10.3389/fnins.2025.1602325 (PMC12728010; doi:10.3389/fnins.2025.1602325)
Supplement: Supplementary file 1 [file Data_Sheet_1.docx]

Supplementary Materials

**Preliminary ERP evidence of the impact of loneliness on Stroop interference for socio-emotional stimuli**

Maria Arioli, Carlotta Maiocchi, Zaira Cattaneo, Claudia Gianelli & Nicola Canessa

**
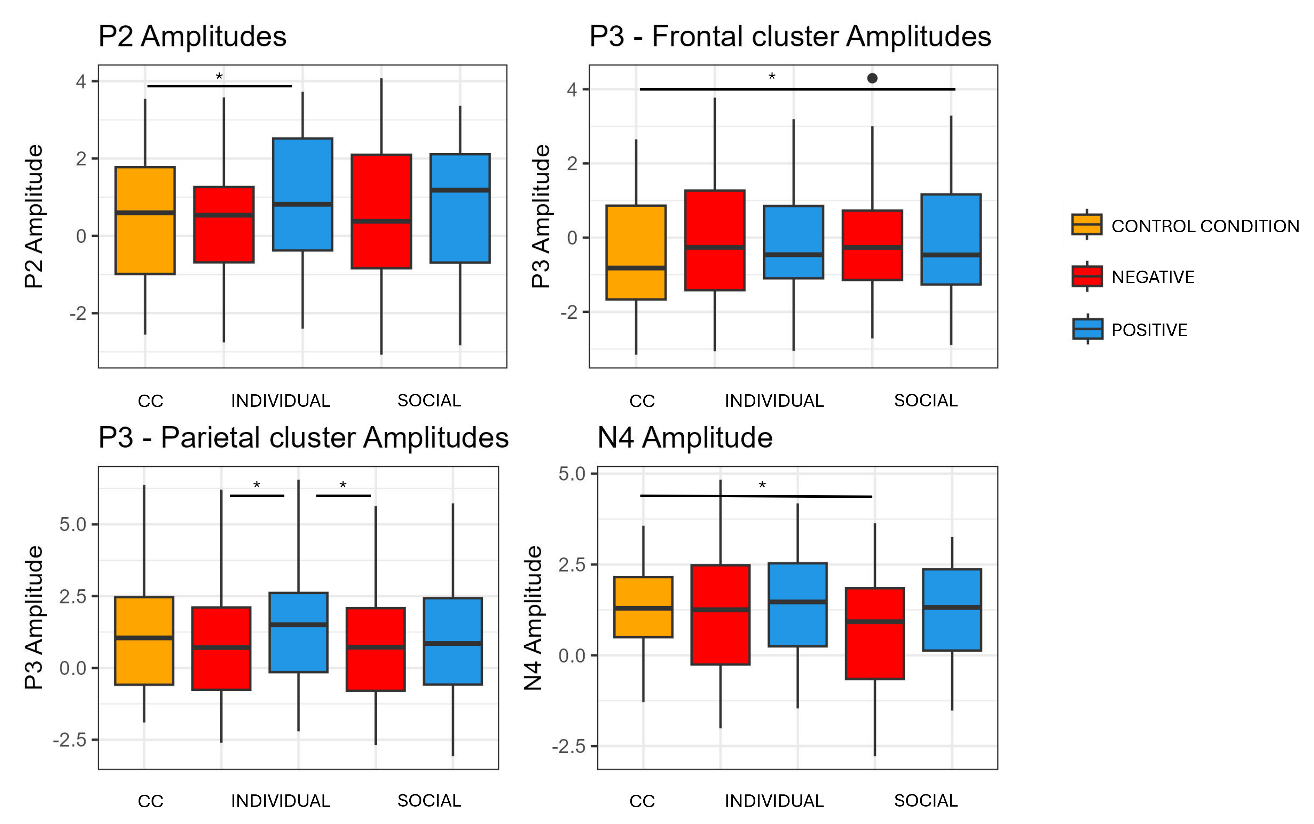
**

**Figure S1.** **ERP component amplitudes across conditions in a socio-emotional Stroop task.** Boxplots depict the distribution of amplitudes for the P2, P3 (frontal and parietal clusters), and N4 components across five experimental conditions involving individual positive, individual negative, social positive, social negative, and control word stimuli. As shown by asterisks, a significant difference in P2 amplitude was observed between the individual positive and control conditions. For the P3 component, the frontal cluster exhibited a significant difference between the social positive and control conditions, while, in the parietal cluster, individual positive words elicited significantly more positive amplitudes compared to both individual negative and social negative words. Additionally, the N4 component showed a significantly more negative modulation in response to social negative, compared to control-neutral, words.
